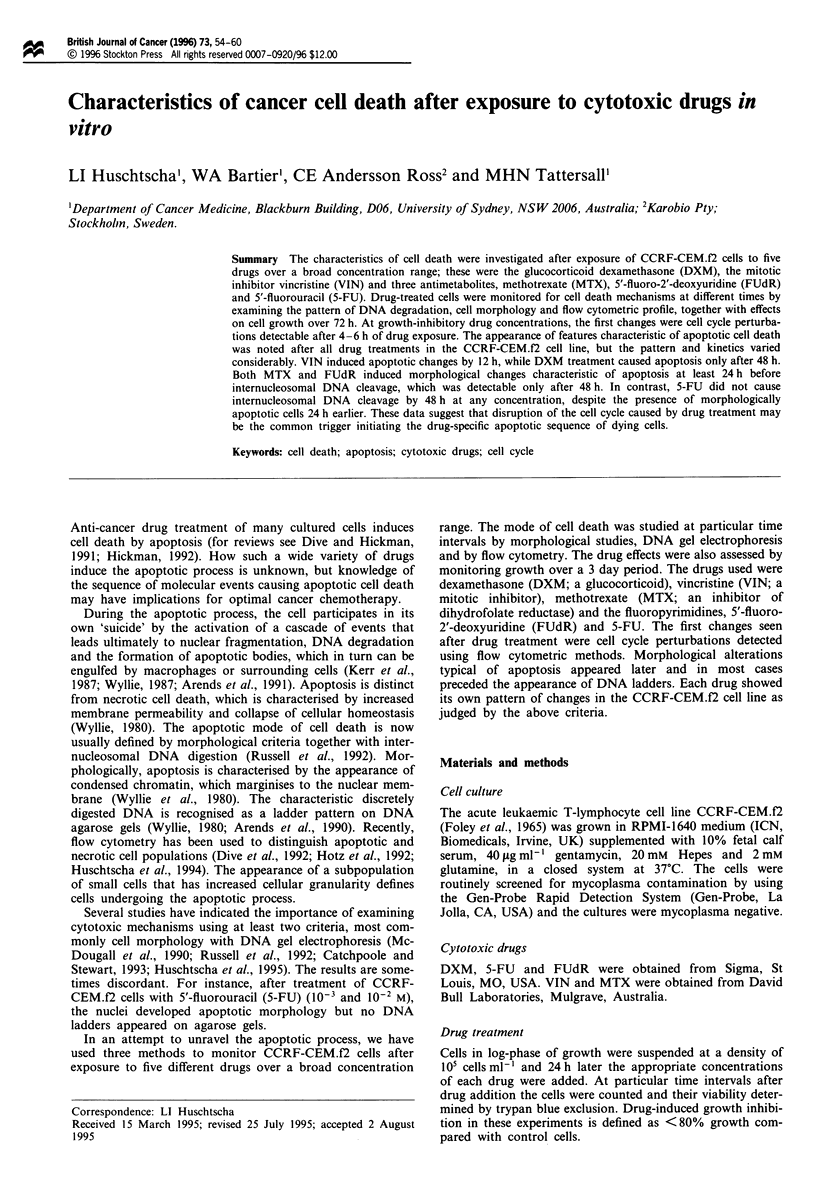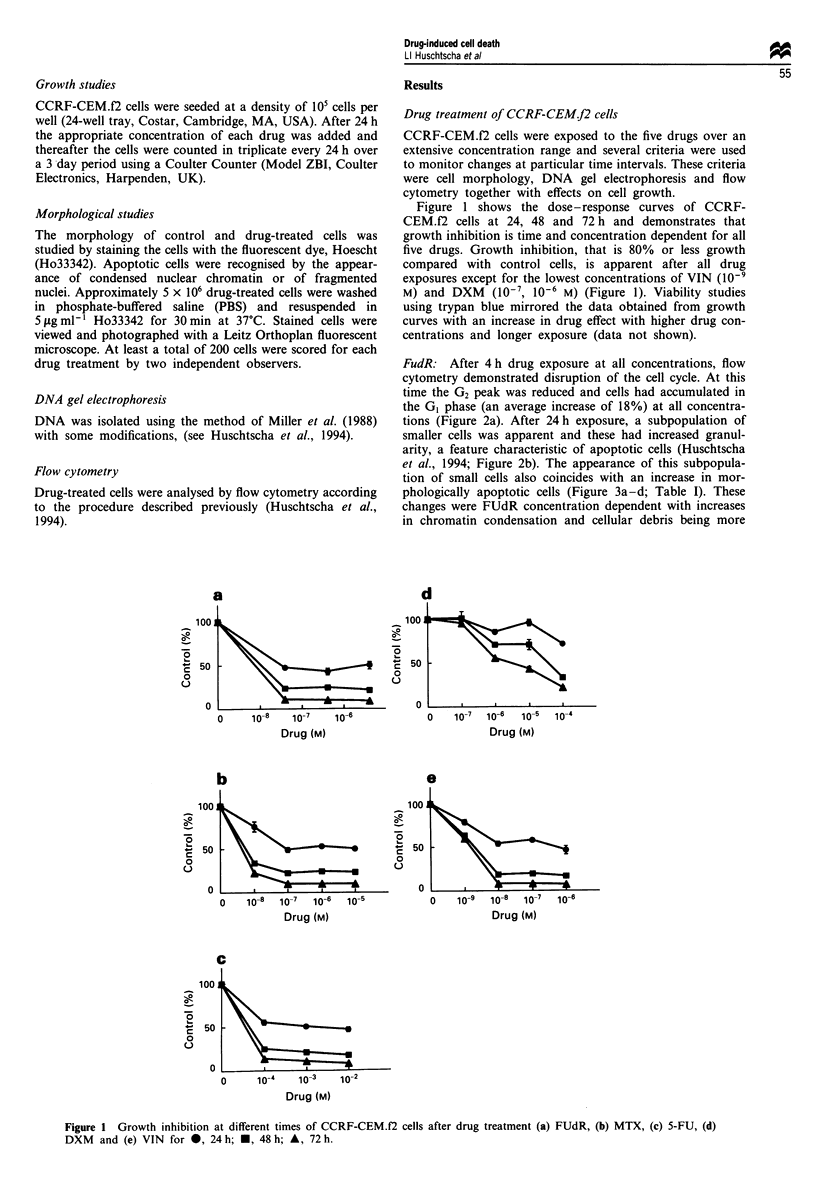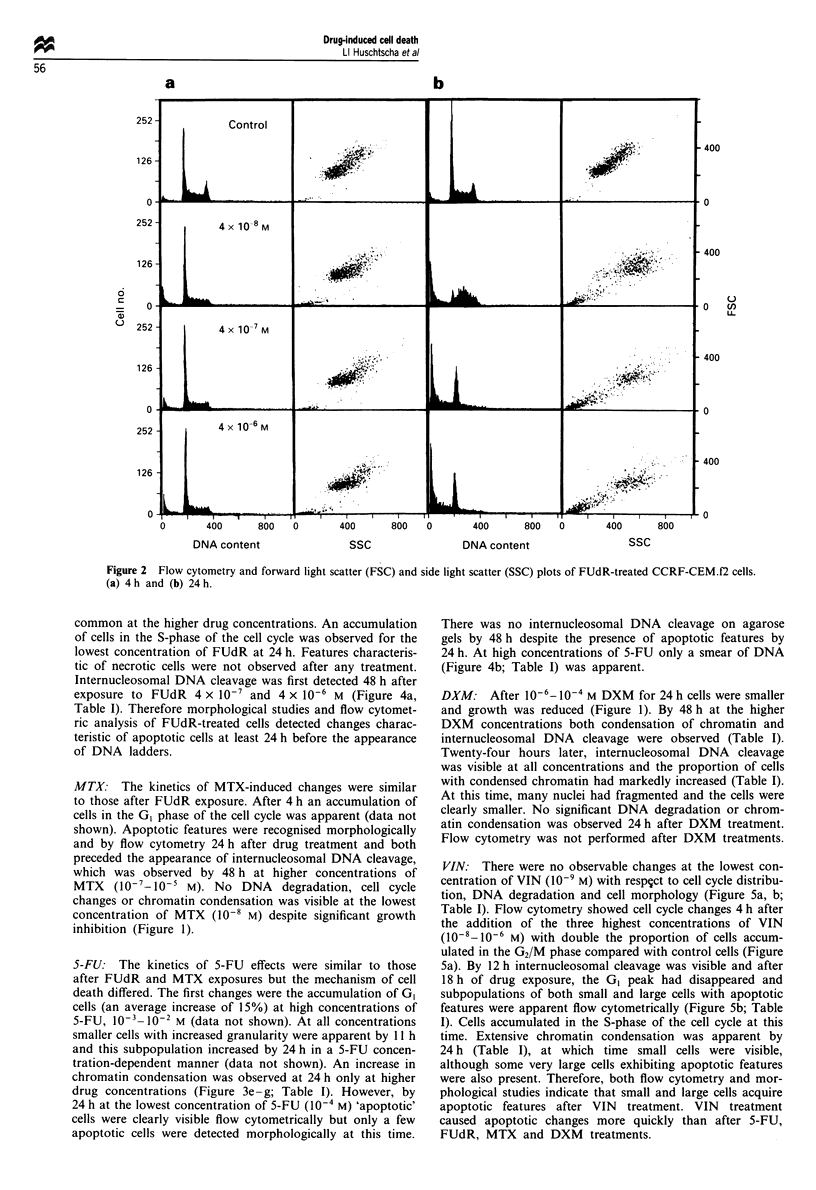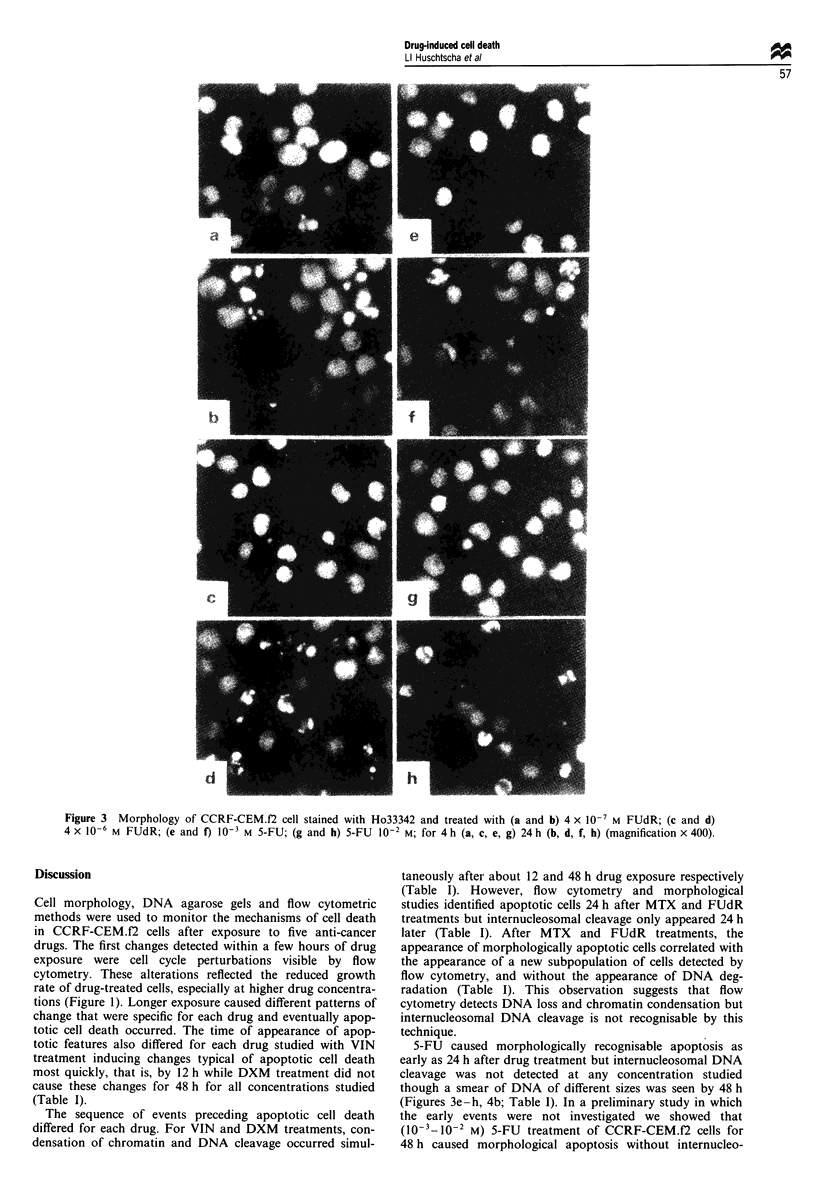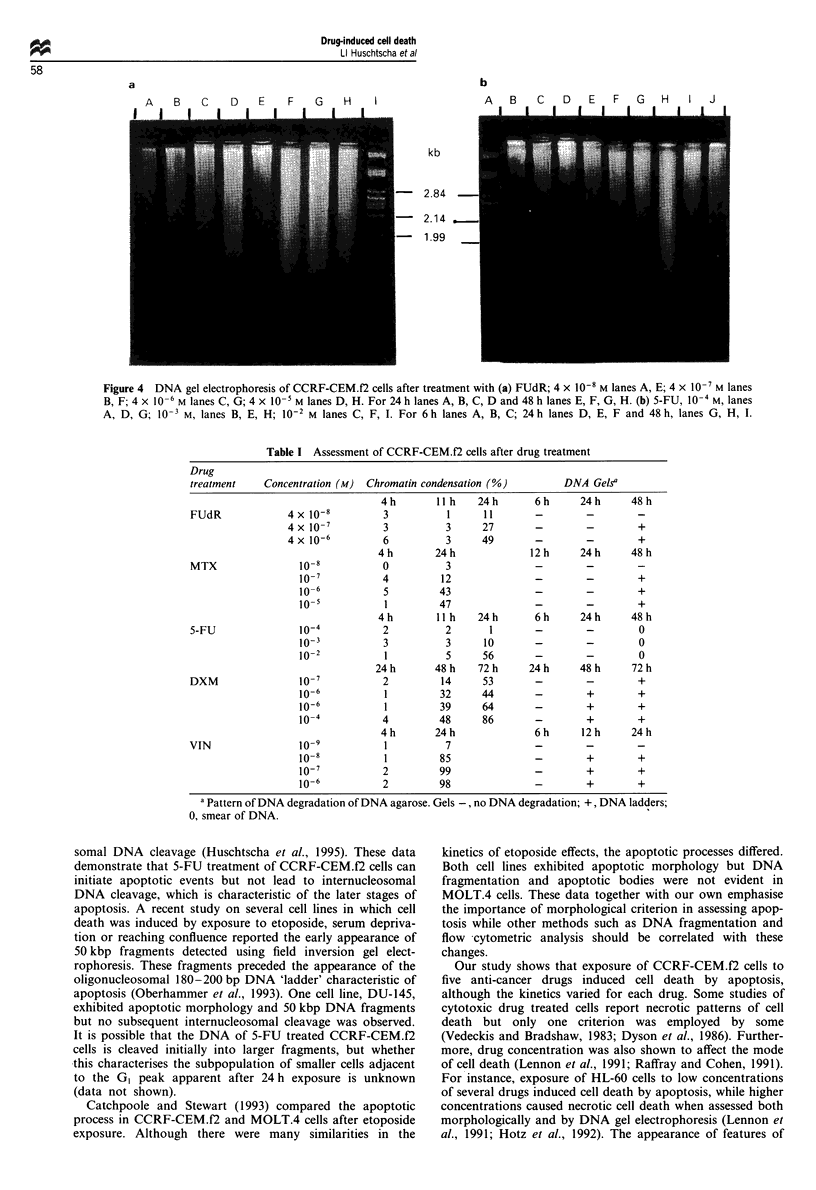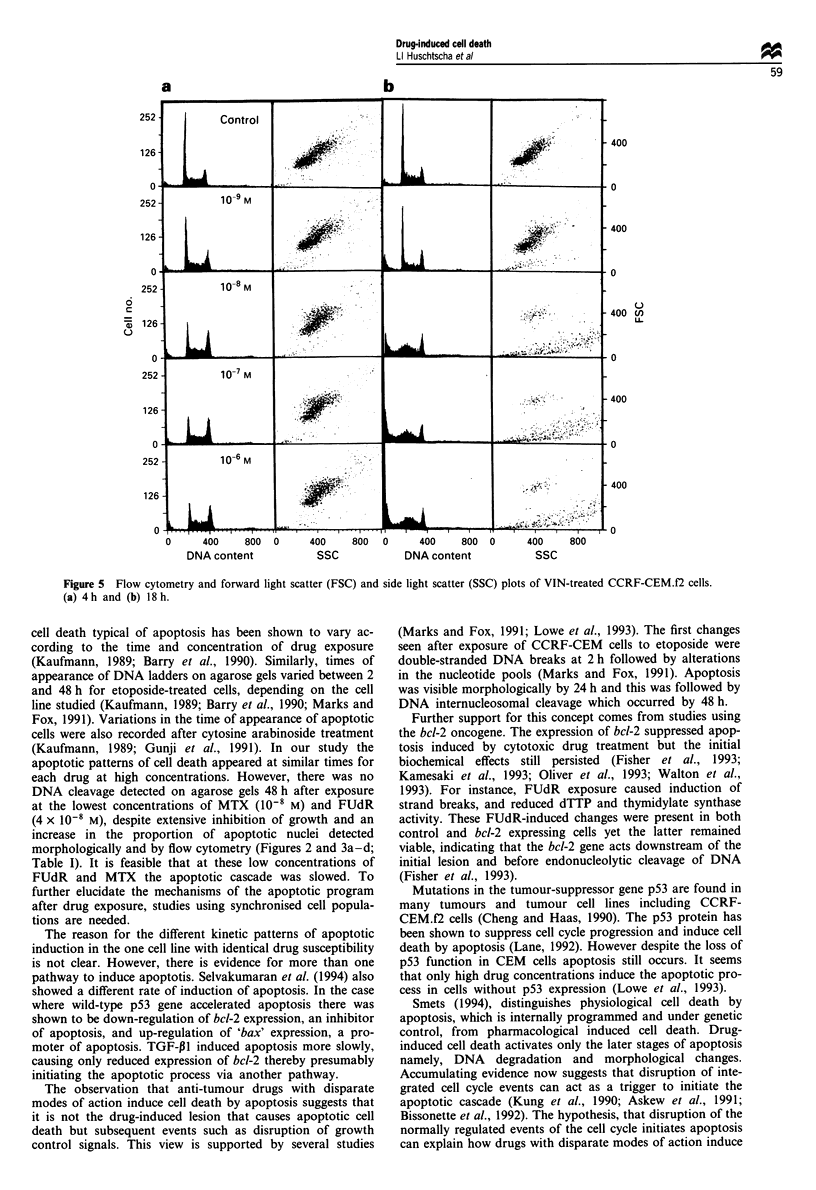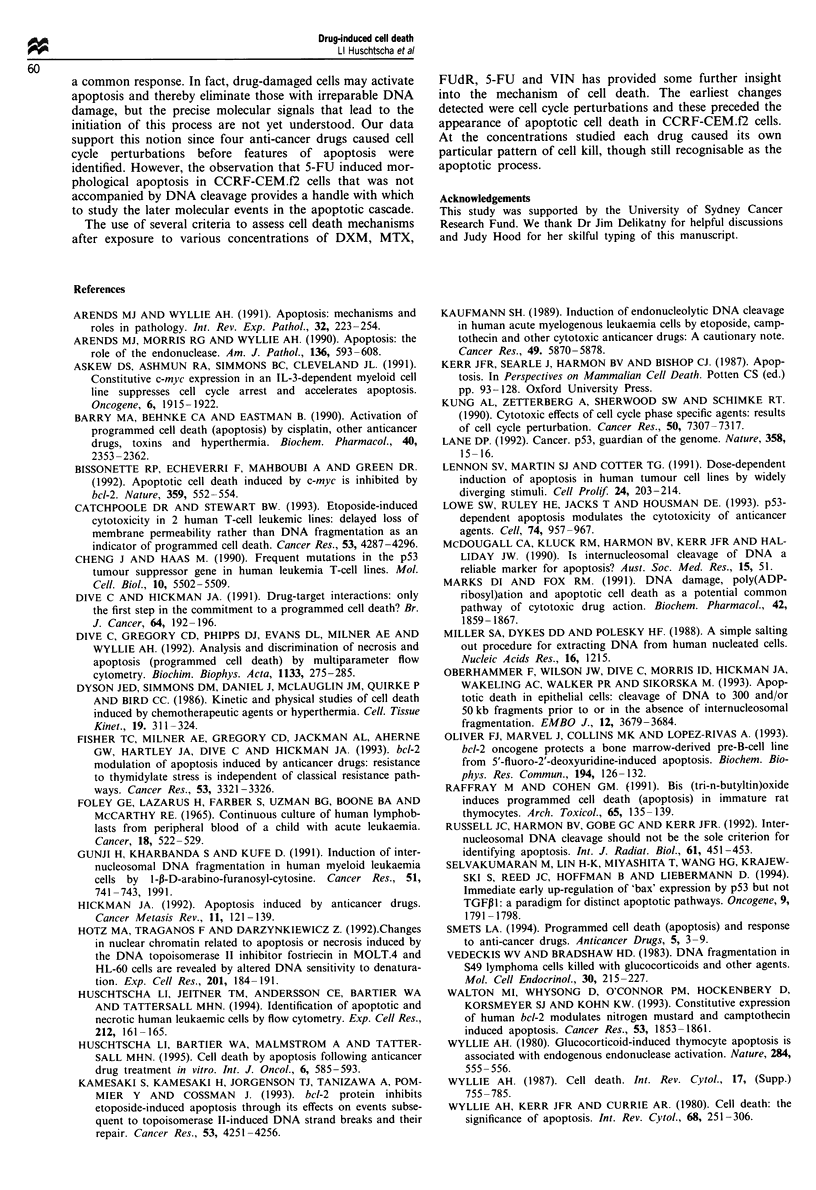# Characteristics of cancer cell death after exposure to cytotoxic drugs in vitro.

**DOI:** 10.1038/bjc.1996.10

**Published:** 1996-01

**Authors:** L. I. Huschtscha, W. A. Bartier, C. E. Ross, M. H. Tattersall

**Affiliations:** Department of Cancer Medicine, University of Sydney, NSW, Australia.

## Abstract

**Images:**


British Journal of Cancer (1996) 73, 54-60
© 1996 Stockton Press All rights reserved 0007-0920/96 $12.00

### Characteristics of cancer cell death after exposure to cytotoxic drugs *in vitro*

LI Huschtscha[1], WA Bartier[1], CE Andersson Ross[2] and MHN Tattersall[1]

[1]*Department of Cancer Medicine, Blackburn Building, D06, University of Sydney, NSW 2006, Australia;* [2]*Karobio Pty; Stockholm, Sweden.*

**Summary** The characteristics of cell death were investigated after exposure of CCRF-CEM.f2 cells to five drugs over a broad concentration range; these were the glucocorticoid dexamethasone (DXM), the mitotic inhibitor vincristine (VIN) and three antimetabolites, methotrexate (MTX), 5'-fluoro-2'-deoxyuridine (FUdR) and 5'-fluorouracil (5-FU). Drug-treated cells were monitored for cell death mechanisms at different times by examining the pattern of DNA degradation, cell morphology and flow cytometric profile, together with effects on cell growth over 72 h. At growth-inhibitory drug concentrations, the first changes were cell cycle perturbations detectable after 4-6 h of drug exposure. The appearance of features characteristic of apoptotic cell death was noted after all drug treatments in the CCRF-CEM.f2 cell line, but the pattern and kinetics varied considerably. VIN induced apoptotic changes by 12 h, while DXM treatment caused apoptosis only after 48 h. Both MTX and FUdR induced morphological changes characteristic of apoptosis at least 24 h before internucleosomal DNA cleavage, which was detectable only after 48 h. In contrast, 5-FU did not cause internucleosomal DNA cleavage by 48 h at any concentration, despite the presence of morphologically apoptotic cells 24 h earlier. These data suggest that disruption of the cell cycle caused by drug treatment may be the common trigger initiating the drug-specific apoptotic sequence of dying cells.

**Keywords:** cell death; apoptosis; cytotoxic drugs; cell cycle

Anti-cancer drug treatment of many cultured cells induces cell death by apoptosis (for reviews see Dive and Hickman, 1991; Hickman, 1992). How such a wide variety of drugs induce the apoptotic process is unknown, but knowledge of the sequence of molecular events causing apoptotic cell death may have implications for optimal cancer chemotherapy.

During the apoptotic process, the cell participates in its own 'suicide' by the activation of a cascade of events that leads ultimately to nuclear fragmentation, DNA degradation and the formation of apoptotic bodies, which in turn can be engulfed by macrophages or surrounding cells (Kerr *et al.*, 1987; Wyllie, 1987; Arends *et al.*, 1991). Apoptosis is distinct from necrotic cell death, which is characterised by increased membrane permeability and collapse of cellular homeostasis (Wyllie, 1980). The apoptotic mode of cell death is now usually defined by morphological criteria together with internucleosomal DNA digestion (Russell *et al.*, 1992). Morphologically, apoptosis is characterised by the appearance of condensed chromatin, which marginises to the nuclear membrane (Wyllie *et al.*, 1980). The characteristic discretely digested DNA is recognised as a ladder pattern on DNA agarose gels (Wyllie, 1980; Arends *et al.*, 1990). Recently, flow cytometry has been used to distinguish apoptotic and necrotic cell populations (Dive *et al.*, 1992; Hotz *et al.*, 1992; Huschtscha *et al.*, 1994). The appearance of a subpopulation of small cells that has increased cellular granularity defines cells undergoing the apoptotic process.

Several studies have indicated the importance of examining cytotoxic mechanisms using at least two criteria, most commonly cell morphology with DNA gel electrophoresis (McDougall *et al.*, 1990; Russell *et al.*, 1992; Catchpoole and Stewart, 1993; Huschtscha *et al.*, 1995). The results are sometimes discordant. For instance, after treatment of CCRF-CEM.f2 cells with 5'-fluorouracil (5-FU) ($10^{-3}$ and $10^{-2}$ M), the nuclei developed apoptotic morphology but no DNA ladders appeared on agarose gels.

In an attempt to unravel the apoptotic process, we have used three methods to monitor CCRF-CEM.f2 cells after exposure to five different drugs over a broad concentration range. The mode of cell death was studied at particular time intervals by morphological studies, DNA gel electrophoresis and by flow cytometry. The drug effects were also assessed by monitoring growth over a 3 day period. The drugs used were dexamethasone (DXM; a glucocorticoid), vincristine (VIN; a mitotic inhibitor), methotrexate (MTX; an inhibitor of dihydrofolate reductase) and the fluoropyrimidines, 5'-fluoro-2'-deoxyuridine (FUdR) and 5-FU. The first changes seen after drug treatment were cell cycle perturbations detected using flow cytometric methods. Morphological alterations typical of apoptosis appeared later and in most cases preceded the appearance of DNA ladders. Each drug showed its own pattern of changes in the CCRF-CEM.f2 cell line as judged by the above criteria.

Correspondence: LI Huschtscha
Received 15 March 1995; revised 25 July 1995; accepted 2 August 1995

### Materials and methods

### *Cell culture*

The acute leukaemic T-lymphocyte cell line CCRF-CEM.f2 (Foley *et al.*, 1965) was grown in RPMI-1640 medium (ICN, Biomedicals, Irvine, UK) supplemented with 10% fetal calf serum, 40 $\mu$g ml$^{-1}$ gentamycin, 20 mM Hepes and 2 mM glutamine, in a closed system at 37°C. The cells were routinely screened for mycoplasma contamination by using the Gen-Probe Rapid Detection System (Gen-Probe, La Jolla, CA, USA) and the cultures were mycoplasma negative.

### *Cytotoxic drugs*

DXM, 5-FU and FUdR were obtained from Sigma, St Louis, MO, USA. VIN and MTX were obtained from David Bull Laboratories, Mulgrave, Australia.

### *Drug treatment*

Cells in log-phase of growth were suspended at a density of $10^5$ cells ml$^{-1}$ and 24 h later the appropriate concentrations of each drug were added. At particular time intervals after drug addition the cells were counted and their viability determined by trypan blue exclusion. Drug-induced growth inhibition in these experiments is defined as <80% growth compared with control cells.

### Growth studies

CCRF-CEM.f2 cells were seeded at a density of $10^5$ cells per well (24-well tray, Costar, Cambridge, MA, USA). After 24 h the appropriate concentration of each drug was added and thereafter the cells were counted in triplicate every 24 h over a 3 day period using a Coulter Counter (Model ZBI, Coulter Electronics, Harpenden, UK).

### Morphological studies

The morphology of control and drug-treated cells was studied by staining the cells with the fluorescent dye, Hoescht (Ho33342). Apoptotic cells were recognised by the appearance of condensed nuclear chromatin or of fragmented nuclei. Approximately $5 \times 10^6$ drug-treated cells were washed in phosphate-buffered saline (PBS) and resuspended in $5\,\mu g\,ml^{-1}$ Ho33342 for 30 min at 37°C. Stained cells were viewed and photographed with a Leitz Orthoplan fluorescent microscope. At least a total of 200 cells were scored for each drug treatment by two independent observers.

### DNA gel electrophoresis

DNA was isolated using the method of Miller *et al.* (1988) with some modifications, (see Huschtscha *et al.*, 1994).

### Flow cytometry

Drug-treated cells were analysed by flow cytometry according to the procedure described previously (Huschtscha *et al.*, 1994).

### Results

### Drug treatment of CCRF-CEM.f2 cells

CCRF-CEM.f2 cells were exposed to the five drugs over an extensive concentration range and several criteria were used to monitor changes at particular time intervals. These criteria were cell morphology, DNA gel electrophoresis and flow cytometry together with effects on cell growth.

Figure 1 shows the dose–response curves of CCRF-CEM.f2 cells at 24, 48 and 72 h and demonstrates that growth inhibition is time and concentration dependent for all five drugs. Growth inhibition, that is 80% or less growth compared with control cells, is apparent after all drug exposures except for the lowest concentrations of VIN ($10^{-9}$ M) and DXM ($10^{-7}$, $10^{-6}$ M) (Figure 1). Viability studies using trypan blue mirrored the data obtained from growth curves with an increase in drug effect with higher drug concentrations and longer exposure (data not shown).

*FudR:* After 4 h drug exposure at all concentrations, flow cytometry demonstrated disruption of the cell cycle. At this time the $G_2$ peak was reduced and cells had accumulated in the $G_1$ phase (an average increase of 18%) at all concentrations (Figure 2a). After 24 h exposure, a subpopulation of smaller cells was apparent and these had increased granularity, a feature characteristic of apoptotic cells (Huschtscha *et al.*, 1994; Figure 2b). The appearance of this subpopulation of small cells also coincides with an increase in morphologically apoptotic cells (Figure 3a–d; Table I). These changes were FUdR concentration dependent with increases in chromatin condensation and cellular debris being more

**Figure 1** Growth inhibition at different times of CCRF-CEM.f2 cells after drug treatment (**a**) FUdR, (**b**) MTX, (**c**) 5-FU, (**d**) DXM and (**e**) VIN for ●, 24 h; ■, 48 h; ▲, 72 h.

**Figure 2** Flow cytometry and forward light scatter (FSC) and side light scatter (SSC) plots of FUdR-treated CCRF-CEM.f2 cells. (**a**) 4 h and (**b**) 24 h.

common at the higher drug concentrations. An accumulation of cells in the S-phase of the cell cycle was observed for the lowest concentration of FUdR at 24 h. Features characteristic of necrotic cells were not observed after any treatment. Internucleosomal DNA cleavage was first detected 48 h after exposure to FUdR $4 \times 10^{-7}$ and $4 \times 10^{-6}$ M (Figure 4a, Table I). Therefore morphological studies and flow cytometric analysis of FUdR-treated cells detected changes characteristic of apoptotic cells at least 24 h before the appearance of DNA ladders.

*MTX:* The kinetics of MTX-induced changes were similar to those after FUdR exposure. After 4 h an accumulation of cells in the $G_1$ phase of the cell cycle was apparent (data not shown). Apoptotic features were recognised morphologically and by flow cytometry 24 h after drug treatment and both preceded the appearance of internucleosomal DNA cleavage, which was observed by 48 h at higher concentrations of MTX ($10^{-7}$–$10^{-5}$ M). No DNA degradation, cell cycle changes or chromatin condensation was visible at the lowest concentration of MTX ($10^{-8}$ M) despite significant growth inhibition (Figure 1).

*5-FU:* The kinetics of 5-FU effects were similar to those after FUdR and MTX exposures but the mechanism of cell death differed. The first changes were the accumulation of $G_1$ cells (an average increase of 15%) at high concentrations of 5-FU, $10^{-3}$–$10^{-2}$ M (data not shown). At all concentrations smaller cells with increased granularity were apparent by 11 h and this subpopulation increased by 24 h in a 5-FU concentration-dependent manner (data not shown). An increase in chromatin condensation was observed at 24 h only at higher drug concentrations (Figure 3e–g; Table I). However, by 24 h at the lowest concentration of 5-FU ($10^{-4}$ M) 'apoptotic' cells were clearly visible flow cytometrically but only a few apoptotic cells were detected morphologically at this time. There was no internucleosomal DNA cleavage on agarose gels by 48 h despite the presence of apoptotic features by 24 h. At high concentrations of 5-FU only a smear of DNA (Figure 4b; Table I) was apparent.

*DXM:* After $10^{-6}$–$10^{-4}$ M DXM for 24 h cells were smaller and growth was reduced (Figure 1). By 48 h at the higher DXM concentrations both condensation of chromatin and internucleosomal DNA cleavage were observed (Table I). Twenty-four hours later, internucleosomal DNA cleavage was visible at all concentrations and the proportion of cells with condensed chromatin had markedly increased (Table I). At this time, many nuclei had fragmented and the cells were clearly smaller. No significant DNA degradation or chromatin condensation was observed 24 h after DXM treatment. Flow cytometry was not performed after DXM treatments.

*VIN:* There were no observable changes at the lowest concentration of VIN ($10^{-9}$ M) with respect to cell cycle distribution, DNA degradation and cell morphology (Figure 5a, b; Table I). Flow cytometry showed cell cycle changes 4 h after the addition of the three highest concentrations of VIN ($10^{-8}$–$10^{-6}$ M) with double the proportion of cells accumulated in the $G_2$/M phase compared with control cells (Figure 5a). By 12 h internucleosomal cleavage was visible and after 18 h of drug exposure, the $G_1$ peak had disappeared and subpopulations of both small and large cells with apoptotic features were apparent flow cytometrically (Figure 5b; Table I). Cells accumulated in the S-phase of the cell cycle at this time. Extensive chromatin condensation was apparent by 24 h (Table I), at which time small cells were visible, although some very large cells exhibiting apoptotic features were also present. Therefore, both flow cytometry and morphological studies indicate that small and large cells acquire apoptotic features after VIN treatment. VIN treatment caused apoptotic changes more quickly than after 5-FU, FUdR, MTX and DXM treatments.

**Figure 3** Morphology of CCRF-CEM.f2 cell stained with Ho33342 and treated with (**a** and **b**) $4 \times 10^{-7}$ M FUdR; (**c** and **d**) $4 \times 10^{-6}$ M FUdR; (**e** and **f**) $10^{-3}$ M 5-FU; (**g** and **h**) 5-FU $10^{-2}$ M; for 4 h (**a**, **c**, **e**, **g**) 24 h (**b**, **d**, **f**, **h**) (magnification × 400).

### Discussion

Cell morphology, DNA agarose gels and flow cytometric methods were used to monitor the mechanisms of cell death in CCRF-CEM.f2 cells after exposure to five anti-cancer drugs. The first changes detected within a few hours of drug exposure were cell cycle perturbations visible by flow cytometry. These alterations reflected the reduced growth rate of drug-treated cells, especially at higher drug concentrations (Figure 1). Longer exposure caused different patterns of change that were specific for each drug and eventually apoptotic cell death occurred. The time of appearance of apoptotic features also differed for each drug studied with VIN treatment inducing changes typical of apoptotic cell death most quickly, that is, by 12 h while DXM treatment did not cause these changes for 48 h for all concentrations studied (Table I).

The sequence of events preceding apoptotic cell death differed for each drug. For VIN and DXM treatments, condensation of chromatin and DNA cleavage occurred simultaneously after about 12 and 48 h drug exposure respectively (Table I). However, flow cytometry and morphological studies identified apoptotic cells 24 h after MTX and FUdR treatments but internucleosomal cleavage only appeared 24 h later (Table I). After MTX and FUdR treatments, the appearance of morphologically apoptotic cells correlated with the appearance of a new subpopulation of cells detected by flow cytometry, and without the appearance of DNA degradation (Table I). This observation suggests that flow cytometry detects DNA loss and chromatin condensation but internucleosomal DNA cleavage is not recognisable by this technique.

5-FU caused morphologically recognisable apoptosis as early as 24 h after drug treatment but internucleosomal DNA cleavage was not detected at any concentration studied though a smear of DNA of different sizes was seen by 48 h (Figures 3e–h, 4b; Table I). In a preliminary study in which the early events were not investigated we showed that ($10^{-3}$–$10^{-2}$ M) 5-FU treatment of CCRF-CEM.f2 cells for 48 h caused morphological apoptosis without internucleo-

**Figure 4** DNA gel electrophoresis of CCRF-CEM.f2 cells after treatment with (**a**) FUdR; $4 \times 10^{-8}$ M lanes A, E; $4 \times 10^{-7}$ M lanes B, F; $4 \times 10^{-6}$ M lanes C, G; $4 \times 10^{-5}$ M lanes D, H. For 24 h lanes A, B, C, D and 48 h lanes E, F, G, H. (**b**) 5-FU, $10^{-4}$ M, lanes A, D, G; $10^{-3}$ M, lanes B, E, H; $10^{-2}$ M lanes C, F, I. For 6 h lanes A, B, C; 24 h lanes D, E, F and 48 h, lanes G, H, I.

**Table I** Assessment of CCRF-CEM.f2 cells after drug treatment

| *Drug treatment* | *Concentration (M)* | *Chromatin condensation (%)* | | | *DNA Gels*[a] | | |
|---|---|---|---|---|---|---|---|
| | | 4 h | 11 h | 24 h | 6 h | 24 h | 48 h |
| FUdR | $4 \times 10^{-8}$ | 3 | 1 | 11 | – | – | – |
| | $4 \times 10^{-7}$ | 3 | 3 | 27 | – | – | + |
| | $4 \times 10^{-6}$ | 6 | 3 | 49 | – | – | + |
| | | 4 h | 24 h | | 12 h | 24 h | 48 h |
| MTX | $10^{-8}$ | 0 | 3 | | – | – | – |
| | $10^{-7}$ | 4 | 12 | | – | – | + |
| | $10^{-6}$ | 5 | 43 | | – | – | + |
| | $10^{-5}$ | 1 | 47 | | – | – | + |
| | | 4 h | 11 h | 24 h | 6 h | 24 h | 48 h |
| 5-FU | $10^{-4}$ | 2 | 2 | 1 | – | – | 0 |
| | $10^{-3}$ | 3 | 3 | 10 | – | – | 0 |
| | $10^{-2}$ | 1 | 5 | 56 | – | – | 0 |
| | | 24 h | 48 h | 72 h | 24 h | 48 h | 72 h |
| DXM | $10^{-7}$ | 2 | 14 | 53 | – | – | + |
| | $10^{-6}$ | 1 | 32 | 44 | – | + | + |
| | $10^{-6}$ | 1 | 39 | 64 | – | + | + |
| | $10^{-4}$ | 4 | 48 | 86 | – | + | + |
| | | 4 h | 24 h | | 6 h | 12 h | 24 h |
| VIN | $10^{-9}$ | 1 | 7 | | – | – | – |
| | $10^{-8}$ | 1 | 85 | | – | + | + |
| | $10^{-7}$ | 2 | 99 | | – | + | + |
| | $10^{-6}$ | 2 | 98 | | – | + | + |

[a] Pattern of DNA degradation of DNA agarose. Gels –, no DNA degradation; +, DNA ladders; 0, smear of DNA.

somal DNA cleavage (Huschtscha *et al.*, 1995). These data demonstrate that 5-FU treatment of CCRF-CEM.f2 cells can initiate apoptotic events but not lead to internucleosomal DNA cleavage, which is characteristic of the later stages of apoptosis. A recent study on several cell lines in which cell death was induced by exposure to etoposide, serum deprivation or reaching confluence reported the early appearance of 50 kbp fragments detected using field inversion gel electrophoresis. These fragments preceded the appearance of the oligonucleosomal 180–200 bp DNA 'ladder' characteristic of apoptosis (Oberhammer *et al.*, 1993). One cell line, DU-145, exhibited apoptotic morphology and 50 kbp DNA fragments but no subsequent internucleosomal cleavage was observed. It is possible that the DNA of 5-FU treated CCRF-CEM.f2 cells is cleaved initially into larger fragments, but whether this characterises the subpopulation of smaller cells adjacent to the $G_1$ peak apparent after 24 h exposure is unknown (data not shown).

Catchpoole and Stewart (1993) compared the apoptotic process in CCRF-CEM.f2 and MOLT.4 cells after etoposide exposure. Although there were many similarities in the kinetics of etoposide effects, the apoptotic processes differed. Both cell lines exhibited apoptotic morphology but DNA fragmentation and apoptotic bodies were not evident in MOLT.4 cells. These data together with our own emphasise the importance of morphological criterion in assessing apoptosis while other methods such as DNA fragmentation and flow cytometric analysis should be correlated with these changes.

Our study shows that exposure of CCRF-CEM.f2 cells to five anti-cancer drugs induced cell death by apoptosis, although the kinetics varied for each drug. Some studies of cytotoxic drug treated cells report necrotic patterns of cell death but only one criterion was employed by some (Vedeckis and Bradshaw, 1983; Dyson *et al.*, 1986). Furthermore, drug concentration was also shown to affect the mode of cell death (Lennon *et al.*, 1991; Raffray and Cohen, 1991). For instance, exposure of HL-60 cells to low concentrations of several drugs induced cell death by apoptosis, while higher concentrations caused necrotic cell death when assessed both morphologically and by DNA gel electrophoresis (Lennon *et al.*, 1991; Hotz *et al.*, 1992). The appearance of features of

**Figure 5** Flow cytometry and forward light scatter (FSC) and side light scatter (SSC) plots of VIN-treated CCRF-CEM.f2 cells. (**a**) 4 h and (**b**) 18 h.

cell death typical of apoptosis has been shown to vary according to the time and concentration of drug exposure (Kaufmann, 1989; Barry *et al.*, 1990). Similarly, times of appearance of DNA ladders on agarose gels varied between 2 and 48 h for etoposide-treated cells, depending on the cell line studied (Kaufmann, 1989; Barry *et al.*, 1990; Marks and Fox, 1991). Variations in the time of appearance of apoptotic cells were also recorded after cytosine arabinoside treatment (Kaufmann, 1989; Gunji *et al.*, 1991). In our study the apoptotic patterns of cell death appeared at similar times for each drug at high concentrations. However, there was no DNA cleavage detected on agarose gels 48 h after exposure at the lowest concentrations of MTX ($10^{-8}$ M) and FUdR ($4 \times 10^{-8}$ M), despite extensive inhibition of growth and an increase in the proportion of apoptotic nuclei detected morphologically and by flow cytometry (Figures 2 and 3a–d; Table I). It is feasible that at these low concentrations of FUdR and MTX the apoptotic cascade was slowed. To further elucidate the mechanisms of the apoptotic program after drug exposure, studies using synchronised cell populations are needed.

The reason for the different kinetic patterns of apoptotic induction in the one cell line with identical drug susceptibility is not clear. However, there is evidence for more than one pathway to induce apoptotis. Selvakumaran *et al.* (1994) also showed a different rate of induction of apoptosis. In the case where wild-type p53 gene accelerated apoptosis there was shown to be down-regulation of *bcl*-2 expression, an inhibitor of apoptosis, and up-regulation of '*bax*' expression, a promoter of apoptosis. TGF-$\beta$1 induced apoptosis more slowly, causing only reduced expression of *bcl*-2 thereby presumably initiating the apoptotic process via another pathway.

The observation that anti-tumour drugs with disparate modes of action induce cell death by apoptosis suggests that it is not the drug-induced lesion that causes apoptotic cell death but subsequent events such as disruption of growth control signals. This view is supported by several studies (Marks and Fox, 1991; Lowe *et al.*, 1993). The first changes seen after exposure of CCRF-CEM cells to etoposide were double-stranded DNA breaks at 2 h followed by alterations in the nucleotide pools (Marks and Fox, 1991). Apoptosis was visible morphologically by 24 h and this was followed by DNA internucleosomal cleavage which occurred by 48 h.

Further support for this concept comes from studies using the *bcl*-2 oncogene. The expression of *bcl*-2 suppressed apoptosis induced by cytotoxic drug treatment but the initial biochemical effects still persisted (Fisher *et al.*, 1993; Kamesaki *et al.*, 1993; Oliver *et al.*, 1993; Walton *et al.*, 1993). For instance, FUdR exposure caused induction of strand breaks, and reduced dTTP and thymidylate synthase activity. These FUdR-induced changes were present in both control and *bcl*-2 expressing cells yet the latter remained viable, indicating that the *bcl*-2 gene acts downstream of the initial lesion and before endonucleolytic cleavage of DNA (Fisher *et al.*, 1993).

Mutations in the tumour-suppressor gene p53 are found in many tumours and tumour cell lines including CCRF-CEM.f2 cells (Cheng and Haas, 1990). The p53 protein has been shown to suppress cell cycle progression and induce cell death by apoptosis (Lane, 1992). However despite the loss of p53 function in CEM cells apoptosis still occurs. It seems that only high drug concentrations induce the apoptotic process in cells without p53 expression (Lowe *et al.*, 1993).

Smets (1994), distinguishes physiological cell death by apoptosis, which is internally programmed and under genetic control, from pharmacological induced cell death. Drug-induced cell death activates only the later stages of apoptosis namely, DNA degradation and morphological changes. Accumulating evidence now suggests that disruption of integrated cell cycle events can act as a trigger to initiate the apoptotic cascade (Kung *et al.*, 1990; Askew *et al.*, 1991; Bissonette *et al.*, 1992). The hypothesis, that disruption of the normally regulated events of the cell cycle initiates apoptosis can explain how drugs with disparate modes of action induce a common response. In fact, drug-damaged cells may activate apoptosis and thereby eliminate those with irreparable DNA damage, but the precise molecular signals that lead to the initiation of this process are not yet understood. Our data support this notion since four anti-cancer drugs caused cell cycle perturbations before features of apoptosis were identified. However, the observation that 5-FU induced morphological apoptosis in CCRF-CEM.f2 cells that was not accompanied by DNA cleavage provides a handle with which to study the later molecular events in the apoptotic cascade.

The use of several criteria to assess cell death mechanisms after exposure to various concentrations of DXM, MTX, FUdR, 5-FU and VIN has provided some further insight into the mechanism of cell death. The earliest changes detected were cell cycle perturbations and these preceded the appearance of apoptotic cell death in CCRF-CEM.f2 cells. At the concentrations studied each drug caused its own particular pattern of cell kill, though still recognisable as the apoptotic process.

**Acknowledgements**
This study was supported by the University of Sydney Cancer Research Fund. We thank Dr Jim Delikatny for helpful discussions and Judy Hood for her skilful typing of this manuscript.

### References

ARENDS MJ AND WYLLIE AH. (1991). Apoptosis: mechanisms and roles in pathology. *Int. Rev. Exp. Pathol.*, **32**, 223–254.

ARENDS MJ, MORRIS RG AND WYLLIE AH. (1990). Apoptosis: the role of the endonuclease. *Am. J. Pathol.*, **136**, 593–608.

ASKEW DS, ASHMUN RA, SIMMONS BC, CLEVELAND JL. (1991). Constitutive c-*myc* expression in an IL-3-dependent myeloid cell line suppresses cell cycle arrest and accelerates apoptosis. *Oncogene*, **6**, 1915–1922.

BARRY MA, BEHNKE CA AND EASTMAN B. (1990). Activation of programmed cell death (apoptosis) by cisplatin, other anticancer drugs, toxins and hyperthermia. *Biochem. Pharmacol.*, **40**, 2353–2362.

BISSONETTE RP, ECHEVERRI F, MAHBOUBI A AND GREEN DR. (1992). Apoptotic cell death induced by c-*myc* is inhibited by *bcl*-2. *Nature*, **359**, 552–554.

CATCHPOOLE DR AND STEWART BW. (1993). Etoposide-induced cytotoxicity in 2 human T-cell leukemic lines: delayed loss of membrane permeability rather than DNA fragmentation as an indicator of programmed cell death. *Cancer Res.*, **53**, 4287–4296.

CHENG J AND HAAS M. (1990). Frequent mutations in the p53 tumour suppressor gene in human leukemia T-cell lines. *Mol. Cell. Biol.*, **10**, 5502–5509.

DIVE C AND HICKMAN JA. (1991). Drug-target interactions: only the first step in the commitment to a programmed cell death? *Br. J. Cancer*, **64**, 192–196.

DIVE C, GREGORY CD, PHIPPS DJ, EVANS DL, MILNER AE AND WYLLIE AH. (1992). Analysis and discrimination of necrosis and apoptosis (programmed cell death) by multiparameter flow cytometry. *Biochim. Biophys. Acta*, **1133**, 275–285.

DYSON JED, SIMMONS DM, DANIEL J, MCLAUGLIN JM, QUIRKE P AND BIRD CC. (1986). Kinetic and physical studies of cell death induced by chemotherapeutic agents or hyperthermia. *Cell. Tissue Kinet.*, **19**. 311–324.

FISHER TC, MILNER AE, GREGORY CD, JACKMAN AL, AHERNE GW, HARTLEY JA, DIVE C AND HICKMAN JA. (1993). *bcl*-2 modulation of apoptosis induced by anticancer drugs: resistance to thymidylate stress is independent of classical resistance pathways. *Cancer Res.*, **53**, 3321–3326.

FOLEY GE, LAZARUS H, FARBER S, UZMAN BG, BOONE BA AND MCCARTHY RE. (1965). Continuous culture of human lymphoblasts from peripheral blood of a child with acute leukaemia. *Cancer*, **18**, 522–529.

GUNJI H, KHARBANDA S AND KUFE D. (1991). Induction of internucleosomal DNA fragmentation in human myeloid leukaemia cells by 1-β-D-arabino-furanosyl-cytosine. *Cancer Res.*, **51**, 741–743, 1991.

HICKMAN JA. (1992). Apoptosis induced by anticancer drugs. *Cancer Metasis Rev.*, **11**, 121–139.

HOTZ MA, TRAGANOS F AND DARZYNKIEWICZ Z. (1992).Changes in nuclear chromatin related to apoptosis or necrosis induced by the DNA topoisomerase II inhibitor fostriecin in MOLT.4 and HL-60 cells are revealed by altered DNA sensitivity to denaturation. *Exp. Cell Res.*, **201**, 184–191.

HUSCHTSCHA LI, JEITNER TM, ANDERSSON CE, BARTIER WA AND TATTERSALL MHN. (1994). Identification of apoptotic and necrotic human leukaemic cells by flow cytometry. *Exp. Cell Res.*, **212**, 161–165.

HUSCHTSCHA LI, BARTIER WA, MALMSTROM A AND TATTERSALL MHN. (1995). Cell death by apoptosis following anticancer drug treatment *in vitro*. *Int. J. Oncol.*, **6**, 585–593.

KAMESAKI S, KAMESAKI H, JORGENSON TJ, TANIZAWA A, POMMIER Y AND COSSMAN J. (1993). *bcl*-2 protein inhibits etoposide-induced apoptosis through its effects on events subsequent to topoisomerase II-induced DNA strand breaks and their repair. *Cancer Res.*, **53**, 4251–4256.

KAUFMANN SH. (1989). Induction of endonucleolytic DNA cleavage in human acute myelogenous leukaemia cells by etoposide, camptothecin and other cytotoxic anticancer drugs: A cautionary note. *Cancer Res.*, **49**. 5870–5878.

KERR JFR, SEARLE J, HARMON BV AND BISHOP CJ. (1987). Apoptosis. In *Perspectives on Mammalian Cell Death*. Potten CS (ed.) pp. 93–128. Oxford University Press.

KUNG AL, ZETTERBERG A, SHERWOOD SW AND SCHIMKE RT. (1990). Cytotoxic effects of cell cycle phase specific agents: results of cell cycle perturbation. *Cancer Res.*, **50**, 7307–7317.

LANE DP. (1992). Cancer. p53, guardian of the genome. *Nature*, **358**, 15–16.

LENNON SV, MARTIN SJ AND COTTER TG. (1991). Dose-dependent induction of apoptosis in human tumour cell lines by widely diverging stimuli. *Cell Prolif.* **24**, 203–214.

LOWE SW, RULEY HE, JACKS T AND HOUSMAN DE. (1993). p53-dependent apoptosis modulates the cytotoxicity of anticancer agents. *Cell*, **74**, 957–967.

MCDOUGALL CA, KLUCK RM, HARMON BV, KERR JFR AND HALLIDAY JW. (1990). Is internucleosomal cleavage of DNA a reliable marker for apoptosis? *Aust. Soc. Med. Res.*, **15**, 51.

MARKS DI AND FOX RM. (1991). DNA damage, poly(ADP-ribosyl)ation and apoptotic cell death as a potential common pathway of cytotoxic drug action. *Biochem. Pharmacol.*, **42**, 1859–1867.

MILLER SA, DYKES DD AND POLESKY HF. (1988). A simple salting out procedure for extracting DNA from human nucleated cells. *Nucleic Acids Res.*, **16**, 1215.

OBERHAMMER F, WILSON JW, DIVE C, MORRIS ID, HICKMAN JA, WAKELING AC, WALKER PR AND SIKORSKA M. (1993). Apoptotic death in epithelial cells: cleavage of DNA to 300 and/or 50 kb fragments prior to or in the absence of internucleosomal fragmentation. *EMBO J.*, **12**, 3679–3684.

OLIVER FJ, MARVEL J, COLLINS MK AND LOPEZ-RIVAS A. (1993). *bcl*-2 oncogene protects a bone marrow-derived pre-B-cell line from 5′-fluoro-2′-deoxyuridine-induced apoptosis. *Biochem. Biophys. Res. Commun.*, **194**, 126–132.

RAFFRAY M AND COHEN GM. (1991). Bis (tri-n-butyltin)oxide induces programmed cell death (apoptosis) in immature rat thymocytes. *Arch. Toxicol.*, **65**, 135–139.

RUSSELL JC, HARMON BV, GOBE GC AND KERR JFR. (1992). Internucleosomal DNA cleavage should not be the sole criterion for identifying apoptosis. *Int. J. Radiat. Biol.*, **61**, 451–453.

SELVAKUMARAN M, LIN H-K, MIYASHITA T, WANG HG, KRAJEWSKI S, REED JC, HOFFMAN B AND LIEBERMANN D. (1994). Immediate early up-regulation of 'bax' expression by p53 but not TGFβ1: a paradigm for distinct apoptotic pathways. *Oncogene*, **9**, 1791–1798.

SMETS LA. (1994). Programmed cell death (apoptosis) and response to anti-cancer drugs. *Anticancer Drugs*, **5**, 3–9.

VEDECKIS WV AND BRADSHAW HD. (1983). DNA fragmentation in S49 lymphoma cells killed with glucocorticoids and other agents. *Mol. Cell Endocrinol.*, **30**, 215–227.

WALTON MI, WHYSONG D, O'CONNOR PM, HOCKENBERY D, KORSMEYER SJ AND KOHN KW. (1993). Constitutive expression of human *bcl*-2 modulates nitrogen mustard and camptothecin induced apoptosis. *Cancer Res.*, **53**, 1853–1861.

WYLLIE AH. (1980). Glucocorticoid-induced thymocyte apoptosis is associated with endogenous endonuclease activation. *Nature*, **284**, 555–556.

WYLLIE AH. (1987). Cell death. *Int. Rev. Cytol.*, **17**, (Supp.) 755–785.

WYLLIE AH, KERR JFR AND CURRIE AR. (1980). Cell death: the significance of apoptosis. *Int. Rev. Cytol.*, **68**, 251–306.